# A Combined Manual Annotation and Deep-Learning Natural Language Processing Study on Accurate Entity Extraction in Hereditary Disease Related Biomedical Literature

**DOI:** 10.1007/s12539-024-00605-2

**Published:** 2024-02-10

**Authors:** Dao-Ling Huang, Quanlei Zeng, Yun Xiong, Shuixia Liu, Chaoqun Pang, Menglei Xia, Ting Fang, Yanli Ma, Cuicui Qiang, Yi Zhang, Yu Zhang, Hong Li, Yuying Yuan

**Affiliations:** 1 BGI Research, Shenzhen, 518083 China; 2 Clinical Laboratory of BGI Health, BGI-Shenzhen, Shenzhen, 518083 China; 3 BGI-Wuhan Clinical Laboratories, BGI-Shenzhen, Wuhan, 430074 China

**Keywords:** Natural language processing, Data mining, Name entity recognition, Genomics

## Abstract

We report a combined manual annotation and deep-learning natural language processing study to make accurate entity extraction in hereditary disease related biomedical literature. A total of 400 full articles were manually annotated based on published guidelines by experienced genetic interpreters at Beijing Genomics Institute (BGI). The performance of our manual annotations was assessed by comparing our re-annotated results with those publicly available. The overall Jaccard index was calculated to be 0.866 for the four entity types—gene, variant, disease and species. Both a BERT-based large name entity recognition (NER) model and a DistilBERT-based simplified NER model were trained, validated and tested, respectively. Due to the limited manually annotated corpus, Such NER models were fine-tuned with two phases. The F1-scores of BERT-based NER for gene, variant, disease and species are 97.28%, 93.52%, 92.54% and 95.76%, respectively, while those of DistilBERT-based NER are 95.14%, 86.26%, 91.37% and 89.92%, respectively. Most importantly, the entity type of variant has been extracted by a large language model for the first time and a comparable F1-score with the state-of-the-art variant extraction model tmVar has been achieved.

**Graphical Abstract:**

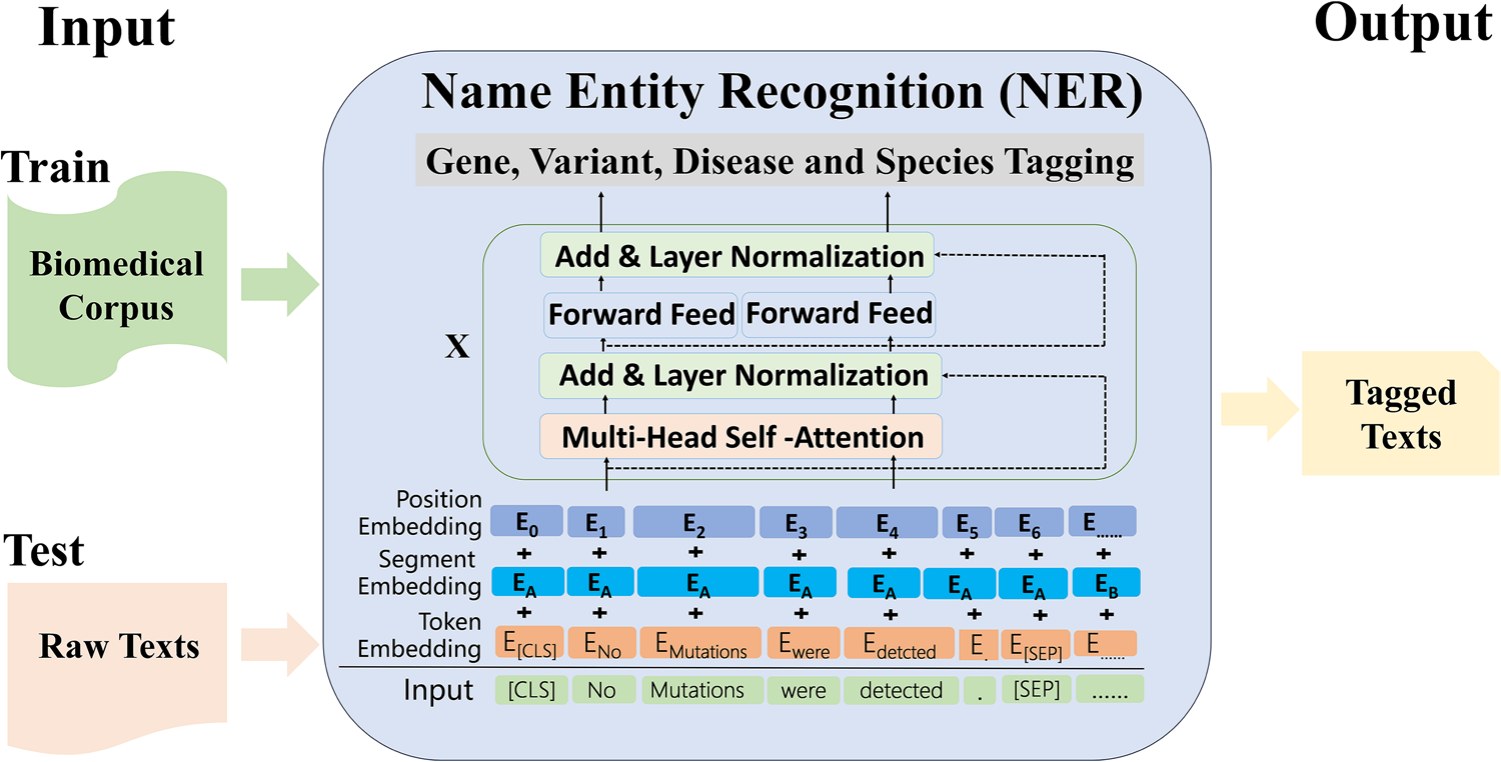

**Supplementary Information:**

The online version contains supplementary material available at 10.1007/s12539-024-00605-2.

## Introduction

With the rapid development of next-generation sequencing technology, the cost of interpreting the clinical significance of hundreds of thousands of genomic variants has become an obvious bottleneck for genetic testing [1–3]. There are dozens of well-established biological databases that are curated and maintained by researchers, which facilitate the interpretation of genomic variants. However, the knowledge provided by these valuable data resources is quite limited [4–9]. Literature in biomedical domain still serve as a huge repository to store tremendous knowledge for genetic variant interpretation. As a result, it poses great challenge for genetic interpreters to search literature manually for relevant evidences for a given variant. For instance, genetic testing and interpretation are pivotal to understand tens of thousands of hereditary diseases. To our best knowledge, although about 90% hereditary disease related variants can be interpretated with public database, the rest 10% hereditary disease related variants still require manual literature evidence search, which is essentially a rate-determining step for genetic variant interpretation. Therefore, it is quite helpful to make literature evidence searching more efficient. In the biomedical domain, one primary application of natural language processing (NLP) is to identify entities in literature [10–13], which is the critical step to develop literature evidence searching tools [14].

As the main tool of entity extraction in free texts, name entity recognition (NER) has long received considerable attention in NLP [10, 15–18]. Web-based services such as Pubtator, LitVar and Pubtator Central (PTC) were launched to automate annotations of literature by combining existing text mining tools that were developed with rule-based and machine-learning-based NER techniques [4, 19–22]. The F1-scores of NER for gene, variant, disease and species that PTC achieved were reported as 86.70%, 86.24%, 83.70% and 85.42%, respectively [19]. It is obvious that there is still room to improve the performance of such techniques due to their limitation of contextualized information [23]. In the past few years, deep-learning neural network (DNN) such as bidirectional long and short term memory (BiLSTM) combined with conditional random field (CRF) have greatly improved performance in NER, but the constraints of sequential computations remain a problem [11–13, 24–26]. In 2018, Google proposed a new self-attention-based language representation model called BERT, which pretrained deep bidirectional representations from unannotated texts and then fine-tuned them on annotated texts [27]. BERT has created state-of-the-art models for a wide range of NLP tasks [27]. In 2019, BioBERT was reported to pretrain and fine-tune pretrained BERT representations on biomedical texts, demonstrating that it is crucial to pretrain BERT on biomedical corpora when applying it to the biomedical domain [28]. However, owing to the scarcity of data with multiple entity types annotated, the fine-tuning stage of BioBERT was trained for each entity type individually. In particular, BioBERT fine-tuning was not applied to variant extraction due to the shortage of variant-annotated corpus, although variant is an extremely important entity type for genetic variant interpretation [28]. In the same year, BioBERT team also reported the web-based tool called BERN to tag entities in PubMed articles or raw texts, relying on tmVar 2.0 to extract variants and BioBERT to extract other entity types such as gene, species, disease and drug [29]. In 2022, BERN was further updated into BERN2 by simply merging its five training sets of all entity types except variant to support parallel inference [30].

In spite of the high performance of BERT-based NER models, training such large models usually consumes quite a few of computing resources and faces significant challenges when it comes to limited computing resources such as on-device real-time applications [31]. A key solution to this problem in artificial intelligence (AI) community is knowledge distillation, in which a small model, so called the student model, is trained to keep the same knowledge of a larger counterpart—the teacher model [31]. There are a bunch of distilled versions of BERT such as BERT-PKD [32], DistilBERT [33], TinyBERT [34], and BERT-EMD [35]. It was reported that DistilBERT reduced the size of a BERT model by 40%, while retaining 97% of its language understanding capabilities and being 60% faster [33]. Furthermore, the interpretability of model distillation can be evaluated using various methods such as comparing the similarity of results between the teacher model and the student model [36].

Here we report a combined manual annotation and deep-learning NLP study to make accurate entity extraction for hereditary disease related biomedical literature, which is a critical step to build a literature evidence tool to interpret the variants associated with hereditary diseases. A total of 400 full biomedical articles were manually annotated based on published guidelines. The interested entity types include gene, variant, disease and species, which are all critical for genetic variant interpretation. The performance of our annotation was evaluated by comparing our re-annotated results with those publicly available [20, 21, 37–40]. Both a BERT-based large model and a DistilBERT-based counterpart were trained and optimized for offline and online inference, respectively. Offline inference refers to the process of generating prediction for all the observations at one time whereas online inference is to handle one observation at a time. The F1-scores of the DistilBERT-based NER model retain 97.8%, 92.2%, 98.7% and 93.9% of those of BERT-based NER for gene, variant, disease and species, respectively. Most importantly, the entity type of variant has been extracted by a large language model for the first time. The three major contributions are summarized as follows:

1. We present a manual annotated dataset of 400 hereditary disease related PubMed full articles for gene, variant, disease and species.
2. We provide an optimized large BERT-basded NER model to extract genes, variants, diseases and species in hereditary disease related biomedical literatures.
3. We provide an optimized small DistilBERT-basded NER version to extract genes, variants, diseases and species in hereditary disease related biomedical literatures.

## Methods

### Annotated Data Acquisition

Annotated literature in this study were obtained in two ways: (1) our manual annotation, (2) downloading from public resources. The manually annotated and downloaded corpora were used for Phase II and Phase I model finetuning, respectively, as is elaborated in 2.3 NER model section. The details about annotated data acquisition are as follows:

#### Our Manual Annotation

Our annotation was focused on four entity types, namely, gene, variant, disease and species. All the annotators have had at least five-year experience in interpreting genetic testing reports at Beijing Genomics Institute (BGI). The annotating procedure was based on published guidelines, namely, GnormPlus/BioCreative II GN for gene, tmVar 2.0 for variant, NCBI disease for disease and Linnaeus for species [20, 21, 38, 39]. There were two major modifications with regards to disease and variant annotation. For one thing, previous experts were encouraged to use their domain knowledge, as well as any other public resources such as UMLS and Wikipedia to annotate disease concepts in NCBI Disease Corpus [38] while our annotators required diseases to be included in professional databases such as Mondo Disease Ontology [41], HPO [42], Orphanet [43], Disease Ontology [44], and OMIM [45]. Particularly, diseases and phenotypes were differentiated to improve the accuracy of annotation. For instance, blistering, nail dystrophy and patchy alopecia in PMID:9457914 were annotated as diseases in NCBI Disease Corpus whereas they were annotated as phenotypes in our corpus because of their records in the HPO database as HP:0008066, HP:0008404, HP:0002232, respectively. For the other thing, while variant concepts in natural language were not annotated but those poorly described were included in tmVarCorpus [21], variants described in free texts were annotated in our corpus as long as specific variants can be obtained such as “Gly in the 163 site was replaced by Ser”. In the cases where there was insufficient information about variants, variants were not annotated to avoid ambiguous descriptions that may mislead subsequent analysis. In order to validate our annotations, part of four publicly available corpora corresponding to gene, variant, disease and species were re-annotated [20, 21, 38, 39]. The details are described in Results Section.

**Fig. 1:**
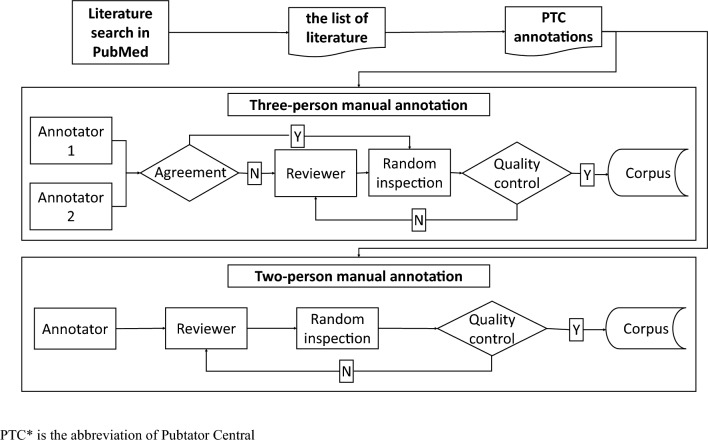
the workflow of manual annotation. PTC* is the abbreviation of Pubtator Central

A total of 400 full hereditary disease related articles on PubMed were annotated. To minimize the variability in annotations by different annotators, the same sample articles were first annotated by all annotators to generate the annotating standards within the team. The workflow of manual annotation is shown in Fig. 1. The whole annotating process was started with literature search on PubMed. There were four steps to retrieve interested articles: (1) Specific genes and the word of “mutation” were searched as key words on https://pubmed.ncbi.nlm.nih.gov. Specific genes referred to the common genes such as SMN1, DMD, PAH and G6PD in common single gene diseases or hereditary cancers. (2) The results were divided into subgroups of five years with the “RESULTS BY YEAR” filter and only full texts were selected with the “Free full text” option in the “TEXT AVAILABILITY” category. (3) Five to eight articles were randomly selected in each subgroup. (4) Only the articles with more than one variant entity were chosen. The interested articles were then annotated with PTC [19], serving as the initial corpus for manual annotation to facilitate our annotating speed. In the early stage of our annotation, the strategy of three-person manual annotation was adopted. Specifically, the same batch of articles were assigned to two annotators independently. If the annotations of a full article agreed with each other, the annotated article would be ready for random inspection; otherwise, the discrepant annotations would go to a reviewer for correction before being added to the batch of articles for random inspection. It is noted that the reviewer also gave the feedback to the original annotators to make sure their annotation strategies would be well aligned over time. In the middle and late stages of our annotation, the strategy of two-person manual annotation was employed for higher efficiency, in which only one annotator and one reviewer were involved for annotating. Subsequently, a certain number of annotated articles in a batch were randomly inspected. The whole batch were aggregated into the annotated corpus only when quality control was completed. In the “two-person manual annotation” strategy, two annotators finished a set of 22 full articles individually and then compared their results. The inter-annotator agreement was 98.3%. In the “three-person manual annotation” strategy, three annotators annotated another set of 14 full articles, reaching 93.1% agreement.

The overall annotating process is still time consuming. On average, it took an annotator 40–45 min to finish one article and 20–25 min to review one article. In practice, the three-person manual annotation and the two-person annotation strategies were implemented on 200 articles, respectively. Therefore, it took approximately 120 h per annotator to annotate all the 400 biomedical articles.

#### BERN-annotated PubMed Abstracts

Due to the small size of our manually annotated corpus, online machine automatically annotated corpora were also downloaded and trained prior to fine-tuning on our manual annotations, as is discussed later in 2.2.1 Section. It is worth noting that the downloaded corpora and our manual annotations should not be overlapped to prevent data leakage between two phases of fine-tuning. The selected dataset was 549,587 BERN-annotated PubMed abstracts in the year of 1978 and 1979 corresponding to pubmed19n0001.json ~ pubmed19n0018.json [29]. These BERN-annotated abstracts did not have overlap with our manually annotated 400 articles which were published after the year of 1990. There were two major reasons why chose the abstracts in the years of 1978 and 1979 instead of choosing among all the years from 1978 to 2019 randomly. On one hand, we believe these two data selection strategies should work similarly because the pretrained model prior to our Phase I fine-tuning model was learned from the abstracts in all the years from 1978 to 2019. This hypothesis was confirmed with the comparison of model metrics between the Phase I models that were built on the abstracts in the years of 1978 and 1979 and those randomly chosen among all the years from 1978 to 2019 (Supplementary Stable 1). On the other hand, practically, the current data selection strategy helps annotators to simplify their future annotating work by avoiding checking if every article they select is overlapped with any of the randomly selected articles. Instead, they only need to avoid the known years. In addition, due to the sparsity of the overall annotated variants in the PubMed abstracts, the sentences containing the entity type of variant in the abstracts spanning the years from 1980 to 2019 but not in our 400 annotated articles were also collected as part of the BERN-annotated PubMed corpus to increase the number of variants.

### Data Preprocessing

Both 549,587 BERN-annotated abstracts and our annotated 400 articles were divided into train/validation/test datasets at the ratio of 7:2:1 randomly for model training, validating and testing for Phase I and Phase II model of BERT, respectively. All the corpora were converted into CoNLL format and labeled using BIO format [46].

### NER Model

**Fig. 2:**
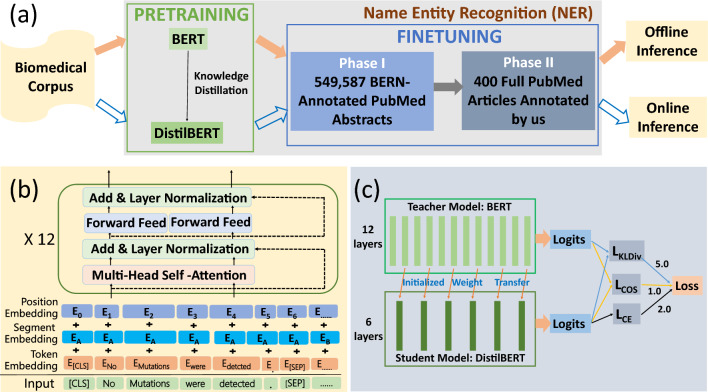
**a** the schematic of BERT-based and DistilBERT-based name entity recognition (NER) pipelines, corresponding to the solid and empty arrows, respectively. The BERT-based model is much larger than the DistilBERT-based model so that the former better fits offline inference while the latter can serve online inference. The NER module contains the steps of pretraining and finetuning with two phases due to the relatively small dataset size for Phase II in the finetuning step. **b** The structure of BERT the inputs are embedded into token vectors and position vectors and then are fed into a 12-layer encoder which consists of multiple self-attention heads. **c** The structural details of knowledge distillation The teacher model (BERT) contains 12 layers while the student model (DistilBERT) has 6 layers. The well pretrained weights of the 2nd, 4th, 6th, 8th, 10th and 12th layers of the teacher model are transferred as the initialized weight of the student model. The output logits of the last layers of both the teacher and student models are used to calculate the total loss of the model according to *Loss* = 5.0 ∗ *L*_*KLDiv*_ + 1.0 ∗ *L*_*COS*_ + 2.0 ∗ *L*_*CE*_

In order to build NER predictive models for both offline inference and online inference, a large model and a compact model were designed correspondingly, namely, BERT-based NER and DistilBERT-based NER, as is shown in Fig. 2a. Both model frameworks contain two steps of training—pretraining and fine-tuning with two phases. Due to the rich knowledge obtained in pretraining, fine-tuning enables models to deal with downstream tasks with limited samples, which is the intuition behind transfer learning. All models in this study were trained, validated and tested on NIVIDIA Quadro RTX 6000 GPUs.

#### BERT-based NER

Our NER model is a pretrained language representation model based on BERT for biomedical literature [28]. The details about the model architecture of BERT were reported [27]. Briefly, BERT is a contextualized word representation model featuring a multi-layer bidirectional Transformer encoder (Fig. 2b) as its major model architecture and a marked language model for pretraining tasks. BioBERT was pretrained on PubMed abstracts and PubMed Central full-text articles using BERT weights as initial model weights [28]. BioBERT was demonstrated to store both general and biomedical knowledge [28].

In our NER model, the weights of pretrained model BioBERT v1.1 were loaded as the pretrained parameters. In the finetuning step, similar to BioBERT, wordpiece embeddings that divide a word into several sub-words were employed so as to recognize both known and out-of-vocabulary words. As is displayed in Fig. 2b, the model architecture is characteristic by twelve encoder layers with self-attention multi-heads. All the encoder layers and self-attention heads have proven to capture different levels of input features such as surface, syntactic and semantic information [27].

However, there were three major modifications of our NER model compared to BioBERT in the fine-tuning step. Firstly, the labels for our NER model included the entity type of variant, which is extremely significant to interpret genetic diseases but was missing in BioBERT due to the shortage of an annotated dataset. Secondly, instead of predicting each entity type separately in BioBERT, our NER model trained all four entity types jointly in a single model, generating representations that captured invariant properties to tasks by sharing features. In order to demonstrate the performance of the joint NER model of the four entity types, both joint modeling and separate modeling were conducted. Finally, instead of only one phase in the fine-tuning step, our NER model consisted of two phases for fine-tuning. That is, the model was first fine-tuned on 549,587 BERN-annotated PubMed abstracts so as to achieve roughly accurate weights before being further fine-tuned on the 400 annotated full articles. The advantage of two phases in fine-tuning of BioBERT was demonstrated by comparing it with the model with one phase that was only fine-tuned on the 400 annotated full articles.

The configuration of the critical training model hyperparameters for our NER model was fixed as follows: the maximum of sequence length, the training batch size, the number of self-attention heads and the learning rate were set to be 512, 8, 12 and 2e-5, respectively. It took about 170 h to train the Phase I fine-tuning step on one NIVIDIA Quadro RTX 6000 GPU.

#### DistilBERT-based NER

General-purpose pretraining distillation was adopted for the purpose of knowledge distillation. As a distilled version of BERT, DistilBERT is characteristic by the overall same architecture as BERT with only half the number of its layers while the token-type embeddings and the pooler are removed [33]. As is seen from Fig. 2c, the architectures of the teacher BERT and the student DistilBERT have twelve and six encoder layers, respectively. The overall training process of DistilBERT-based NER model is the same as that of BERT-based model. However, the student model was initialized from the teacher model by taking the latter one layer out of two and is trained to reproduce the behavior of teacher model. The training loss is given by [33]

1 $${\text{Loss}} = 5.0 \times L_{{{\text{KLDiv}}}} + 1.0 \times L_{{{\text{COS}}}} + 2.0 \times L_{E}$$

where *L*_*KLDiv*_ is  Kullback–Leibler divergence loss between the soft target probabilities of the teacher and the student, *L*_*COS*_ is the cosine embedding loss between the soft target probabilities of the teacher and the student and *L*_*CE*_ is the cross-entropy loss of the soft target probabilities of the student.

The configuration of the critical training model hyperparameters for our DistilBERT-based NER model was selected as the same of those in BERT-based NER model. It took about 40 h to train the Phase I finetuning step on two NIVIDIA Quadro RTX 6000 GPUs, which is over 50% faster than its large counterpart model.

#### Model Validation and Evaluation

In order to achieve a satisfactory performance metric, the hyperparameters of a trained model were tuned on validation dataset. Once model validation was completed, the model was tested with the test dataset to predict and evaluate the performance. Metrics such as entity-level precision, recall and F1 scores of each model were computed for model performance evaluation.

## Results

### Consistency Analysis Between our Annotators and Experts Annotating the Publicly Available Corpora

Table 1 displays the statistics of our annotations, publicly available annotated corpora from experts previously, the intersection and the Jaccard index between these two parties. Specifically, the annotated datasets for gene, variant, disease and species correspond to GnormPlus/BioCreative II GN, tmVar 2.0, NCBI disease and Linnaeus, respectively [20, 21, 38, 39]. In total, 3818 genes, variants, diseases and species were annotated from our annotators while the total number for experts previously is 3868, resulting in an overall Jaccard index of 0.866 between the annotated corpora from our annotators and previous experts, indicating that there is a considerable degree of overlap between the two annotated datasets (Table 2). Specifically, the Jaccard indexes for gene, variant and specie are all over 0.800, among which those for gene and species are both as high as 0.953 while that for disease is 0.688. The list of all the inconsistent cases is provided in Supplementary Stable 2.

**Table 1:** the statistics of our annotations, publicly available annotated corpora from experts previously, the intersection and the Jaccard index between these two parties

Entity Type	Dataset	Number of Annotations			Jaccard Index
		Ours	Experts’	Intersection	
Gene	GnormPlus/BioCreative II GN	1269	1256	1232	0.953
Variant	tmVar 2.0	509	464	437	0.815
Disease	NCBI disease	844	961	736	*0.688*
Species	Linnaeus	1196	1187	1163	0.953
Total		3818	3868	3568	0.866

**Table 2:** The statistics of inconsistent annotated entities between experts previously and our annotators due to three different factors (discrepant rules of both annotating parties, the false annotation from the experts and our false annotation), the total inconsistent number, the false annotation rate of experts and the rate of discrepant rules

Entity type	Inconsistent number due to different factors			Total	Experts’ false annotation rate (#Experts False annotation/ #Total, %)	The rate of discrepant rules (#Discrepant Rules/#Total, %)
	Discrepant rules	Experts’ false annotation	Our false annotation			
Gene	**14**	2	8	24	8.33	58.33
Variant	**26**	1	0	27	3.70	96.30
Species	**19**	3	2	24	12.50	79.17
Disease	**160**	58	7	225	25.78	71.11
Total	219	64	17	300	21.33	73.33

The most significant factor that contributes to annotation inconsistency between experts and our annotators for each entity type is bolded

### The Statistics of Annotated Entities in Datasets in the Fine-tuning Step

The statistics of all and unique annotations in datasets for Phase I and Phase II in the fine-tuning step of NER models are shown in Table 3, corresponding to 549,587 BERN-annotated PubMed abstracts and 400 full articles annotated by us, respectively. Train-ratio, validation-ratio and test-ratio represent the percentages of the numbers of each entity type in train, validation and test datasets, respectively. As is generally accepted, the degrees of imbalance are considered as mild, moderate and extreme when the proportion of a minority class is 20–40%, 1–20% and < 1% of the dataset, respectively. The mild and moderate datasets are often treated as normal datasets while extreme ones need special treatment such as sampling techniques. As is shown in Table 3, all the percentages of the numbers of all the entity types in train, validation and test datasets in both phases suggest either mild or moderate data imbalance. Therefore, all the datasets can be treated normally. In addition, the numbers of all annotations in the train, validation and test datasets in Phase I in the fine-tune step are 30–50 times, 25–50 times and 20–75 times of those in Phase II while the numbers of unique annotations in the train, validation and test datasets in Phase I are 30–130 times, 50–200 times and 75–370 times of those in Phase II, respectively, indicating that the annotated corpus in the fine-tuning step was considerably enriched from our small-size dataset by adding publicly available machine annotated corpora. It is noted that the number of entities of species are smaller than the other three in that all the 400 annotated full articles are human related articles.

**Table 3:** The statistics of annotations in datasets for Phase I and Phase II in the fine-tuning step, corresponding to 549,587 BERN-annotated PubMed abstracts and our 400 annotated full articles, respectively

Number of annotations in fine-tuning						
Phase	Dataset	Statistic method	Entity type			
			Gene	Variant	Disease	Species
Phase I	Train	All	1,002,127	858,759	804,217	596,828
		Unique	253,012	260,248	165,024	37,124
	Train-ratio	All	31%	26%	25%	18%
		Unique	35%	36%	23%	5%
	Validation	All	286,716	249,472	228,937	169,618
		Unique	97,179	101,572	63,490	13,849
	Validation-ratio	All	31%	27%	24%	18%
		Unique	35%	37%	23%	5%
	Test	All	144,811	124,706	116,144	84,247
		Unique	56,520	57,502	38,263	7,799
	Test-ratio	All	31%	27%	25%	18%
		Unique	35%	36%	24%	5%
Phase II	Train	All	33,443	18,117	26,729	16,177
		Unique	2186	8756	3008	380
	Train-ratio	All	35%	19%	28%	17%
		Unique	15%	61%	21%	3%
	Validation	All	11,061	4985	8016	4272
		Unique	512	2184	993	157
	Validation-ratio	All	39%	18%	28%	15%
		Unique	13%	57%	26%	4%
	Test	All	6265	1743	4166	2176
		Unique	152	703	446	99
	Test-ratio	All	44%	12%	29%	15%
		Unique	11%	50%	32%	7%

Train-ratio, validation-ratio and test-ratio represent the percentages of the numbers of each entity type in train, validation and test datasets, respectively. The best metrics of each entity type are bolded

### Performance Comparison of BERT-based NER with One Phase and Two Phases in the Fine-tuning Step

In Table 4, the rows containing BERT (2 phases) and BERT (1 phase) in the joint entity extraction mode correspond to the performances of BERT-based NER to predict gene, variant, disease and species with two phases and one phase in the finetuning step, respectively. BERT (1 phase) represents the model in which only 400 annotated articles were used for fine-tuning. The performance of BERT-based NER in Phase I was validated by comparing it with different biomedical text mining tools such as PTC, Hunflaire [47], BERN and BERN2 (Supplementary Stable 3). It is obvious that the F1-scores of BERT (2 phases) to predict gene, variant, disease and species are 97.28%, 93.52%, 92.54% and 95.76%, which are improved by 0.77%, 2.46%, 0.56% and 1.54% upon the corresponding F1-scores of BERT (1 phase), 96.51%, 91.06%, 91.98% and 94.21%, respectively. This observation suggests that BERT-based NER with two phases in the finetuning step outperforms the one with only one phase. The 2.46% F1-score increase for variant is particularly important because of the critical role that variants usually play in the interpretation of genetic diseases. It seems that BERT (2 phases) has a lower false positive rate than BERT (1 phase) by 5.17% precision value (94.31% versus 89.14%).

**Table 4:** The performance comparison of joint-entity-extraction-mode BERT-based NER with two phases [BERT (2 phases)], DistilBERT-based NER with two phases [DistilBERT (2 phases)], BERT-based NER with one phase [BERT (1 phase)] and single-entity-extraction-mode BERT-based NER with two phases [BERT (2 phases)] in the fine-tuning step for gene, variant, disease and species at the entity level

Entity type	Entity extraction mode	Model	Precision (%)	Recall (%)	F1-Score (%)
Gene	Joint	BERT (2 phases)	97.11	97.45	**97.28**
		DistilBERT (2 phases)	94.84	95.45	95.14
		BERT (1 phase)	95.69	97.35	96.51
	Single	BERT (2 phases)	95.99	96.40	96.19
Variant	Joint	BERT (2 phases)	94.31	92.75	**93.52**
		DistilBERT (2 phases)	85.94	86.59	86.26
		BERT (1 phase)	89.14	93.06	91.06
	Single	BERT (2 phases)	89.94	92.23	91.07
Disease	Joint	BERT (2 phases)	91.22	93.90	**92.54**
		DistilBERT (2 phases)	90.44	92.32	91.37
		BERT (1 phase)	91.54	92.43	91.98
	Single	BERT (2 phases)	91.19	91.60	91.40
Species	Joint	BERT (2 phases)	98.30	93.34	**95.76**
		DistilBERT (2 phases)	96.39	84.26	89.92
		BERT (1 phase)	97.66	90.99	94.21
	Single	BERT (2 phases)	97.66	89.14	93.21

BERT-based NER with one phase [BERT (1 phase)] in the finetuning step means that only the 400 annotated articles were used for finetuning. The best F1-scores of each entity type are bolded

### Performance Comparison of BERT-based NER in Term of Joint and Single Entity Extraction Methods

Both of the rows containing BERT (2 phases) show the performance of BERT-based NER in the finetuning step for gene, variant, disease and species at the entity level in term of joint and single entity extraction modes in Table 4. All the metrics such as F1-score, precision and recall values corresponding to joint entity extraction are higher than those corresponding to single entity extraction, indicating that training all entity types jointly makes it possible to share the features of all interested entities and thus provides much more information than a model that is trained separately. In addition, the comparison of the performance statistics of BERT-based NER in Phase I is provided in Supplementary Stable 4. It is noted that this comparison is less insightful than that in Phase II since the annotations for Phase I modeling are all from BERN, whose F1-scores for gene, variant, disease and species were reported as 84.40%, 93.70%, 89.36% and 89.81%, respectively [29]. The main purpose of Phase I fine-tuning is to help the model learn patterns through valuable information contained in the roughly accurate data.

### Performance Comparison of BERT-based NER and DistilBert-based NER

The rows containing BERT (2 phases) and DistilBERT (2 phases) in Table 4 correspond to the performance of BERT-based NER and DistilBERT-based NER for gene, variant, disease and species at the entity level, respectively. The F1-scores of NER of gene, variant, disease and species for the BERT-based model are 97.28%, 93.52%, 92.54% and 95.76%, respectively, while those for the DistilBERT-based model are 95.14%, 86.26%, 91.37% and 89.92%, respectively. Therefore, F1 scores of the DistilBERT-based NER model retain 97.8%, 92.2%, 98.7% and 93.9% of those of BERT-based NER for gene, variant, disease and species, respectively. This observation demonstrated the effectiveness of knowledge distillation of DistilBERT (2 phases) from BERT (2 phases). Similar to the BERT-based NER model, the performance of DistilBERT in Phase I was also validated by comparing with different lightweighted models such as DistilBERT, DistilBioBERT, CompactBioBERT and TinyBioBERT [48], as is provided in Supplementary Stable 5.

## Case Study

While NER models were reported to have general applications including discovery of new named entities, information retrieval and relation extraction [29], we developed the NER models for hereditary disease related literature for one more specific purpose of genetic interpretation of hereditary disease. That is, we proposed to construct an American College of Medical Genetics (ACMG) recommendation based evidence knowledge graph for hereditary disease. The recommendation of ACMG developed a set of criteria to weight variant evidence and a set of rules for combining criteria to arrive at one of the five classification tiers [49]. Genetic counselors often need to read literature and interpret the variants based on ACMG recommendation manually, which is quite time consuming. NER for hereditary disease literatures holds promise to accelerate genetic interpretation. For example, the criterion of PP1 (Tier 1) in ACMG recommendation is “Cosegregation with disease in multiple affected family members in a gene definitively known to cause the disease”, which can be described in the form of a triplet (variant, cosegregate, PP1) of the knowledge graph. We used BERT-based NER model to text mine the article (PMID:29271107) and identified the sentence containing a variant “The novel c.1232G > A is a truncating and function disrupting mutation of the CHEK2 gene, identified in an early onset breast cancer proband.” By searching the key word “cosegregate*” in its preceding, current and subsequent sentences, the evidence of “The high number of breast cancers observed in this family, cosegregation of the variant with the disease and its LOH in the breast cancer tissue, strongly suggest this is a breast cancer predisposing allele.” was spotted. Therefore, the conclusion that c.1232G > A is a pathogenic variant is supported by PP1 criterion. In this way, a comprehensive ACMG evidence knowledge graph can be constructed to automate the interpretation as much as possible.

## Discussion

Supervised deep learning usually depends on the datasets with high quality. Problematic cases that are often unpredictable, not well-represented or outliers of the majority of the data pose significant challenges such as misclassification or prediction errors, generalization issues and reduced model robustness. However, in practice, it is often difficult to obtain such ideal corpus due to the limitation of knowledge of annotators. In this work, experienced genetic interpreters in hereditary diseases at BGI were selected as annotators. The very few false annotations we observed in the datasets of gene, variant and species annotated by previous experts validated the reliability of these datasets. However, there were many more false annotations in the previously annotated disease dataset, suggesting that there is room to improve this dataset. Moreover, the fact that discrepant rules are the dominant factor that caused the inconsistency between our annotations and those from previous experts actually implied different purposes of the annotations. It seems that previous experts annotated for general audience while ours emphasized on accuracy for professional purposes such as genetic interpretation.

Supervised deep learning also highly depends on the datasets with large quantity but is often limited by the labor of annotators. Our study well demonstrated that annotating biomedical literature was both time consuming and knowledge-intensive. Meanwhile, it also proved that it was feasible to insert one more finetuning phase trained with roughly accurately annotated corpus before a very accurately annotated but small one. Although the roughly accurate data may introduce random noise and uncertainty to the model, it can also be very helpful to supplement considerable label information to improve the predictive ability of the model, especially when the model is robust.

Notably, our manually annotated corpus enabled us to train all the four entity types jointly instead of being limited by scattered annotated corpora that were publicly available, as the challenge BioBERT was faced. In addition to the convenience to perform a downstream task within one model instead of separating the task into sub-tasks for sub-models, an obvious advantage is that the joint model can be easily extended to relationship extraction tasks where at least two entity types should be included. It is also noted that our manually annotated corpus enables us to extract the entity type of variant using a large language model for the first time. Due to the shortage of a high-quality public dataset of variants, BERN used tmVar 2.0 as a variant NER model and the reported F1-score was 93.70% [29]. The key to achieve a high F1-score of tmvar 2.0 was to apply regular expression rules in the post-processing step after CRF modeling. Our study demonstrated that BERT-based NER model without applying regular expression rules had comparable predictive ability of machine learning and rule-based modeling.

Furthermore, training deep-learning NN models often requires tremendous resources and time. Fortunately, pretrained models based on huge corpora are often readily reused in NLP community. For instance, in order to adapt BERT to biomedical texts, BioBERT was re-pretrained with PubMed abstracts and PubMed central full-text articles based on the BERT pretrained model [28]. We started our model by loading pretrained BioBERT v1.1 model weights and distilled the  model to transfer the knowledge of the teacher model to the student model, which significantly reduced the number of parameters in the student model, thereby reducing the storage space and computational resources required by the model. The well-trained DistilBERT-based NER model should be able to be applied to online inference. In practice, online inference can be used to build interactive prediction tools while offline inference is applied to large knowledge base construction.

Several directions for future work can be proposed based on this study. Firstly, automated algorithms can be explored to accelerate the process since manually annotating is a time-consuming process. Secondly, a highly accurate web-based platform for entity tagging is likely to be built by applying optimized BERT-based NER models to a large number of literature. Thirdly, the DistilBERT-based NER model can be used for real-time entity extraction on a web-based tagging platform. The last but not the least, the model framework in this study is essentially supervised learning of a very specific biomedical field, which poses great challenge for generalizing the model to a broader field. In near future, a large foundation model coupled with limited human feedback reinforcement learning can be attempted to solve the generalization problem.

## Conclusions

We report a combined manual annotation and deep-learning NLP study to make accurate NER for biomedical literature. A total of 400 full articles from PubMed were annotated. Both a BERT-based large model and a DistilBERT-based simplified model were constructed, trained and optimized for offline and online inference, respectively. Both of them outperform the state-of-art model—BioBERT, indicating the significance to train an NER model on biomedical literature jointly with annotated datasets. It is quite promising for the models to be applied to the construction of a useful and efficient entity-tagging platform.

### Supplementary Information

Below is the link to the electronic supplementary material.

Supplementary file1 (PDF 665 KB)

## Data Availability

All the annotated train, validate and test datasets are available at https://github.com/dlhuang/Entity_Extraction_Hereditary_Disease_2023/tree/master/data.

## Code availability

Demo source codes are available at https://github.com/bgimedx/biomed_ner.
