# Supplementary material for: A Combined Manual Annotation and Deep-Learning Natural Language Processing Study on Accurate Entity Extraction in Hereditary Disease Related Biomedical Literature
Source: Interdiscip Sci. 2024 Feb 10;16(2):333–44. doi: 10.1007/s12539-024-00605-2 (PMC11289304; doi:10.1007/s12539-024-00605-2)
Supplement: Supplementary file 1 — Supplementary file1 (PDF 665 KB) [file 12539_2024_605_MOESM1_ESM.pdf]

Journal: Interdisciplinary Sciences: Computational Life Sciences

A Combined Manual Annotation and Deep-learning Natural Language Processing Study on Accurate Entity Extraction in Hereditary Disease Related Biomedical Literature

Dao-Ling Huang[1,2*], Quanlei Zeng[3], Yun Xiong[3], Shuixia Liu[3], Chaoqun Pang[3], Menglei Xia[3], Ting Fang[3], Yanli Ma[3], Cuicui Qiang[3], Yi Zhang[3], Yu Zhang[3], Hong Li[3] and Yuying Yuan[2]

[1] BGI Research, Shenzhen 518083, China

[2]Clinical Laboratory of BGI Health, BGI-Shenzhen, Shenzhen, 518083, China

[3]BGI-Wuhan Clinical Laboratories, BGI-Shenzhen, WuHan, 430074, China

[*]dlhuang1217@gmail.com

Stable 1. The comparison of model metrics between the Phase I models that were built on the abstracts in the years of 1978 and 1979 and those randomly chosen among all the years from 1978 to 2019.

Entity Type	Entity Extraction Method	BERT Phase I			
		Precision (%)	Recall (%)	F1-Score (%)	F1-Score (%) Joint_Random-
**Gene**	**Joint**	87.56	88.25	87.90	0.21
	**Joint_Random**	88.01	88.21	**88.11**	
**Variant**	**Joint**	85.76	98.15	91.54	0.08
	**Joint_Random**	85.89	98.18	**91.62**	
**Disease**	**Joint**	90.17	91.64	**90.90**	-0.13
	**Joint_Random**	90.19	91.37	90.77	
**Species**	**Joint**	97.20	98.42	97.81	0.10
	**Joint_Random**	97.33	98.49	**97.91**	

Gene						
PMID	Pos_Start	Pos_End	Our Annotatior	Experts' Annotation	Detailed Information	Classification
10949293	587	617	-	selectively methylate lysine 9	The entity is not a gene or protein and should not be annotated.	Experts' False
11152476	1093	1103	-	PDZ domain	The entity is a protein domain and should not be annotated.	
10766764	175	236	beta2	calcium-activated K(+) channel beta-subunits, beta2 and beta3	The entity is the full name of a gene and should be annotated as a whole.	Our False
10766764			beta3			
10882741	77	133	ELL3	testis-specific RNA polymerase II elongation		
11056056	1550	1553	-	Sp1	The entity is a gene and should be annotated.	
11056056	1568	1572	-	AP-1		
11056056	1578	1581	-	Pu1		
11165367	794	800	-	p47MNR		
11980910	1885	1889	-	MKK3		
10458166	36	39	-	BMP	The entity is a gene or protein group and should not be annotated according to our annotation guidelines.	Discrepancy Rules
10458166	706	732	-	bone morphogenetic protein		
10458166	734	737	-	BMP		
10458166	798	806	-	TGF-beta		
10458166	1202	1205	-	BMP		
10458166	1464	1467	-	BMP		
10458166	1582	1590	-	TGF-beta		
10458166	1732	1740	-	TGF-beta		
11152476	175	177	-	L1		
11152476	194	208	-	immunoglobulin		
11152476	790	792	-	L1		
11152476	810	812	-	L1		
11781389	449	461	-	receptor of C1q		
11781389	757	760	-	C1q		

The "-" indicates that we did not annotate the entity.
The "Pos_Start" and "Pos_End" were provided by the corresponding corpus.

Variant						
PMID	Pos_Start	Pos_End	Our Annotatior	Experts' Annotation	Detailed Information	Classification
14751036	1594	1601	intron 9 +1G>T	9 +1G>T	The entity is a variant and should be fully annotated.	Experts' False
15820770	335	344	GAT-->GAG substitution replaces aspartic acid by glutamic acid in codon 416	GAT-->GAG	The entity is a variant described by natural language and should be fully annotated according to our annotation guidelines.	Discrepancy Rules
15579915	1176	1186	sequence variant located in the intron 10 of the RET oncogene as an A to G substitution denoted IVS10 + 4G	IVS10 + 4G		
20738799	577	580	-	T>C	The entity cannot determine the specific variant and should not be annotated according to our annotation guidelines.	
17968299	491	494	-	G/A		
17961316	688	693	-	G > C		
16001362	858	863	-	C-->T		
15754732	1460	1467	-	GAC/GAT		
21135151	14	21	-	delta32		
17909797	263	270	-	Delta32		
17909797	845	852	-	Delta32		
17909797	1134	1141	-	Delta32		
17909797	1553	1560	-	Delta32		
17166870	491	496	-	6bINS		
17166870	642	647	-	6bINS		
17166870	1119	1124	-	6bINS		
17166870	1418	1423	-	6bINS		
17166870	1552	1557	-	6bINS		
15461822	356	363	-	Delta30		
15461822	573	580	-	Delta30		
15461822	842	849	-	Delta30		
15461822	1083	1090	-	Delta30		
15461822	1262	1269	-	Delta30		
15461822	1287	1294	-	Delta30		
15461822	1445	1452	-	Delta30		
15461822	1495	1502	-	Delta30		
15461822	1512	1519	-	Delta30		

The "-" indicates that we did not annotate the entity.
The "Pos_Start" and "Pos_End" were provided by the corresponding corpus.

Disease							
PMID	Text	Pos_Start	Pos_End	Our Annotation	Experts' Annotation	Detailed Inforn	lassificatio

9448273	<category ="Specific Disease"> VHL</category>	683	725	-	VHL		
9448273	<category ="Modifier ">VHL</category>	1026	1061	-	VHL		
9448273	<category ="Specific Disease"> VHL</category>	1444	1486	-	VHL		
9448273	<category ="Modifier ">VHL</category>	1577	1612	-	VHL		
9448273	<category ="Specific Disease"> VHL</category>	1814	1856	-	VHL		
9448273	<category ="Specific Disease"> VHL</category>	1871	1913	-	VHL		
9585611	<category ="Modifier ">APC</category>	903	938	-	APC		
9585611	<category ="Modifier ">APC</category>	1107	1142	-	APC		
9585611	<category ="Modifier ">APC</category>	1246	1281	-	APC		
9585611	<category ="Modifier ">APC</category>	2260	2295	-	APC		
9618166	<category ="Modifier ">PDS</category>	585	620	-	PDS		
9618166	<category ="Modifier ">PDS</category>	1439	1474	-	PDS		
9618170	<category ="Modifier ">DMD</category>	57	92	-	DMD		
9618170	<category ="Modifier ">Duchenne muscular dystrophy </category >	608	667	-	Duchenne muscular dystrophy		
9702690	<category ="Specific Disease"> ALD</category>	263	305	-	ALD		
9724771	<category ="Modifier ">APC</category>	4	39	-	APC		
9724771	<category ="Modifier ">APC</category>	559	594	-	APC		
9724771	<category ="Modifier ">APC</category>	809	844	-	APC		
9724771	<category ="Modifier ">APC</category>	1233	1268	-	APC		
9724771	<category ="Specific Disease"> APC</category>	1420	1462	-	APC		

9724771	<category ="Modifier">APC</category>	1844	1879	-	APC		
9724771	<category ="Modifier">APC</category>	2296	2331	-	APC		
9729124	<category ="Modifier">congenital chloride diarrhea</category>	31	91	-	congenital chloride diarrhea		
9729124	<category ="Modifier">CLD</category>	94	129	-	CLD		
9729124	<category ="Modifier">CLD</category>	395	430	-	CLD		
9729124	<category ="Modifier">CLD</category>	1121	1156	-	CLD	The entity is a gene and should not be annotated as a disease.	
9731533	<category ="Specific Disease">APC</category>	608	650	-	APC		
9733027	<category ="Modifier">FRDA</category>	145	181	-	FRDA		
9733027	<category ="Specific Disease">Friedreich ataxia</category>	187	243	-	Friedreich ataxia		
9733027	<category ="Modifier">FRDA</category>	327	363	-	FRDA		
9770531	<category ="Modifier">von Hippel-Lindau</category>	133	182	-	von Hippel-Lindau		Experts' False
9770531	<category ="Modifier">von Hippel-Lindau</category>	224	273	-	von Hippel-Lindau		
9770531	<category ="Modifier">VHL</category>	276	311	-	VHL		
9770531	<category ="Specific Disease">VHL</category>	842	884	-	VHL		
9770531	<category ="Modifier">VHL</category>	1160	1195	-	VHL		
9800909	<category ="Modifier">DMD</category>	704	739	-	DMD		
9800909	<category ="Modifier">DMD</category>	1504	1539	-	DMD		
9863607	<category ="Modifier">DM</category>	722	756	-	DM		
9863607	<category ="Modifier">DM</category>	1493	1527	-	DM		
9869602	<category ="Modifier">APC</category>	25	60	-	APC		

9869602	<category ="Modifier ">APC</category>	380	415	-	APC		
9950360	<category ="Modifier ">APC</category>	26	61	-	APC		
9950360	<category ="Modifier ">APC</category>	200	235	-	APC		
9950360	<category ="Modifier ">adenomatous polyposis coli</category>	635	693	-	adenomatous polyposis coli		
9950360	<category ="Modifier ">APC</category>	696	731	-	APC		
9950360	<category ="Modifier ">APC</category>	799	834	-	APC		
9950360	<category ="Modifier ">APC</category>	972	1007	-	APC		
9950360	<category ="Modifier ">APC</category>	1101	1136	-	APC		
9973276	<category ="Modifier ">APC</category>	199	234	-	APC		
9973276	<category ="Modifier ">APC</category>	542	577	-	APC		
9973276	<category ="Modifier ">APC</category>	686	721	-	APC		
9465301	<category ="Specific Disease">uniparental disomy</category>	286	343	chromosome 15 paternal uniparental disomy	uniparental disomy	The entity is the full name of a disease and should be annotated as a whole.	
9585605	<category ="Specific Disease">uniparental disomy</category>	269	326	paternal uniparental disomy (UPD) 15	uniparental disomy		
9585605	<category ="Specific Disease"> UPD</category>	329	371		UPD		
9973276	<category ="Specific Disease">Inherited colorectal polyposis</category>	0	69	colorectal polyposis and canc	Inherited colorectal polyposis		
9973276	<category ="Modifier ">cancer</category>	74	112		cancer		
9744473	<category ="Specific Disease"> Deficiency of arylsulfatase A</category>	121	189	-	Deficiency of arylsulfatase A	The entity is the cause of a	

9888388	<category ="Specific Disease">deficient activity of coproporphyrinogen III oxidase</category>	377	468	-	deficient activity of coproporphyrinogen III oxidase	disease and should not be annotated.	
932197	<category ="Disease Class">pyogenic infection</category>	2543	2597	-	pyogenic infection	The entity is a disease and should be annotated.	Our False
9674906	<category ="Specific Disease">contractures</category>	866	917	-	contractures		
9703418	<category ="Specific Disease">meningococcal meningitis</category>	550	613	-	meningococcal meningitis		
9790667	<category ="Specific Disease">deficiency in the sterol 27-hydroxylase activity</category>	1462	1549	-	deficiency in the sterol 27-hydroxylase activity		
9949209	<category ="Specific Disease">WD</category>	1694	1735	-	WD	The entity is the abbreviation of a disease and should be annotated.	
9949209	<category ="Specific Disease">CT</category>	1760	1801	-	CT		
9949209	<category ="Specific Disease">CT</category>	1887	1928	-	CT		
9288106	<category ="Disease Class">recessive multi-system disorder</category>	282	349	-	recessive multi-system disorder		
9288106	<category ="Disease Class">cancer</category>	425	467	-	cancer		
9288106	<category ="Modifier">tumour</category>	686	724	-	tumour		
9288106	<category ="Disease Class">clonal malignancy</category>	886	939	-	clonal malignancy		
9288106	<category ="Modifier">tumour</category>	1880	1918	-	tumour		
9288106	<category ="Modifier">tumour</category>	2068	2106	-	tumour		

9288106	<category="DiseaseClass">tumours</category>	2451	2494	-	tumours
9288106	<category="Modifier">tumour</category>	2650	2688	-	tumour
9311732	<category="Modifier">tumor</category>	223	260	-	tumor
9311732	<category="Modifier">tumor</category>	688	725	-	tumor
9311732	<category="DiseaseClass">tumors</category>	751	793	-	tumors
9311732	<category="DiseaseClass">tumors</category>	1003	1045	-	tumors
9311732	<category="DiseaseClass">tumors</category>	1199	1241	-	tumors
9311732	<category="DiseaseClass">tumors</category>	1273	1315	-	tumors
9311732	<category="DiseaseClass">tumors</category>	1383	1425	-	tumors
9311732	<category="DiseaseClass">tumors</category>	1518	1560	-	tumors
9311732	<category="DiseaseClass">tumors</category>	2267	2309	-	tumors
932197	<category="DiseaseClass">bacterial infections</category>	2208	2264	-	bacterial infections
9342365	<category="Modifier">tumor</category>	280	317	-	tumor
9342365	<category="Modifier">tumor</category>	1493	1530	-	tumor
9358014	<category="DiseaseClass">cancer</category>	1232	1274	-	cancer
9360520	<category="DiseaseClass">inherited disease</category>	318	371	-	inherited disease
9360520	<category="DiseaseClass">dominantly inherited neurodegeneration</category>	1422	1496	-	dominantly inherited neurodegeneration

9371490	<category ="Disease Class">male cancer</category>	188	235	-	male cancer
9371490	<category ="Disease Class">cancer</category>	344	386	-	cancer
9371490	<category ="Modifier">tumor</category>	718	755	-	tumor
9371490	<category ="Disease Class">tumors</category>	1372	1414	-	tumors
9385378	<category ="Disease Class">genetic defect</category>	378	428	-	genetic defect
9391889	<category ="Disease Class">muscular weakness</category>	364	417	-	muscular weakness
9420335	<category ="Modifier">tumor</category>	4	41	-	tumor
9420335	<category ="Modifier">tumor</category>	183	220	-	tumor
9420335	<category ="Disease Class">Growth retardation</category>	588	642	-	Growth retardation
9439660	<category ="Disease Class">malignancy</category>	517	563	-	malignancy
9439660	<category ="Modifier">cancer</category>	682	720	-	cancer
9439660	<category ="CompositeMention">familial and sporadic cancers</category>	1580	1649	-	familial and sporadic cancers
9439660	<category ="Modifier">tumor</category>	1722	1759	-	tumor
9448273	<category ="Modifier">cancer</category>	870	908	-	cancer
9448273	<category ="Modifier">tumor</category>	1711	1748	-	tumor
9448273	<category ="Modifier">tumor</category>	1927	1964	-	tumor
9457914	<category ="Disease Class">dental abnormalities</category>	590	646	-	dental abnormalities

9457914	<category="Disease Class">Skin fragility</category>	649	699	-	Skin fragility
9467011	<category="Modifier">tumour</category>	268	306	-	tumour
9467011	<category="Disease Class">malignancy</category>	505	551	-	malignancy
9467011	<category="Disease Class">tumours</category>	727	770	-	tumours
9521421	<category="Disease Class">genetic defect</category>	428	478	-	genetic defect
9529364	<category="Disease Class">urogenital abnormalities</category>	1203	1263	-	urogenital abnormalities
9585583	<category="Disease Class">autosomal dominant condition</category>	244	308	-	autosomal dominant condition
9585583	<category="Disease Class">limb anomalies</category>	372	422	-	limb anomalies
9585583	<category="Disease Class">craniosynostotic condition</category>	1755	1817	-	craniosynostotic condition
9585611	<category="Modifier">tumor</category>	139	176	-	tumor
9600235	<category="Disease Class">recessive disease</category>	315	368	-	recessive disease
9618170	<category="Disease Class">myocardial involvement</category>	419	477	-	myocardial involvement
9620771	<category="Disease Class">adrenal insufficiency</category>	67	124	-	adrenal insufficiency
9620771	<category="Disease Class">genetic defect</category>	1773	1823	-	genetic defect

9620771	<category="Disease Class">adrenal insufficiency</category>	2001	2058	-	adrenal insufficiency	The entity is the name of a type of diseases or not recorded in our reference databases and should not be annotated according to our annotation guidelines.	
9634518	<category="Disease Class">allelic disorders</category>	438	491	-	allelic disorders		
9668171	<category="Disease Class">genetic diseases</category>	831	883	-	genetic diseases		
9671401	<category="Modifier">tumor</category>	137	174	-	tumor		
9671401	<category="Disease Class">unilateral tumors</category>	450	503	-	unilateral tumors		
9671401	<category="Disease Class">tumors</category>	937	979	-	tumors		
9674903	<category="Disease Class">Maternal disomy</category>	0	51	-	Maternal disomy		
9674903	<category="Disease Class">Maternal disomy </category>	1610	1662	-	Maternal disomy		
9674903	<category="Disease Class">Uniparental disomy</category>	1995	2049	-	Uniparental disomy		
9674906	<category="Disease Class">neuromuscular disorder</category>	1470	1528	-	neuromuscular disorder		
9674906	<category="Specific Disease"> bone dysplasia</category>	2044	2097	-	bone dysplasia		
9702690	<category="Disease Class">abnormalities in the bilateral cerebellar hemispheres</category>	1058	1147	-	abnormalities in the bilateral cerebellar hemispheres		
9702690	<category="Disease Class">demyelination of the cerebral white matter</category>	1403	1481	-	demyelination of the cerebral white matter		

9702690	<category ="Disease Class">insidious lesion</category>	1986	2038	-	insidious lesion
9731533	<category ="Modifier">cancer</category>	25	63	-	cancer
9731533	<category ="Disease Class">polyps</category>	402	444	-	polyps
9731533	<category ="Disease Class">tumours</category>	708	751	-	tumours
9731533	<category ="Disease Class">cancer</category>	1181	1223	-	cancer
9770531	<category ="Disease Class">cancers</category>	1455	1498	-	cancers
9770531	<category ="Modifier">cancer</category>	1672	1710	-	cancer
9771706	<category ="Disease Class">autosomal recessive neurodegenerative disorder</category>	363	445	-	autosomal recessive neurodegenerative disorder
9774970	<category ="Modifier">tumor</category>	63	100	-	tumor
9792860	<category ="Disease Class">glomerular basement membrane abnormalities</category>	341	419	-	glomerular basement membrane abnormalities
9792861	<category ="Modifier">cancer</category>	488	526	-	cancer
9800909	<category ="Disease Class">cerebral dysfunction</category>	1336	1392	-	cerebral dysfunction
9831355	<category ="Disease Class">cancer</category>	773	815	-	cancer
9861003	<category ="Disease Class">genetic disorders</category>	1094	1147	-	genetic disorders
9861003	<category ="Disease Class">affective disorders</category>	2090	2145	-	affective disorders
9869602	<category ="Disease Class">cancer</category>	1258	1300	-	cancer

9869602	<category="Modifier">cancer</category>	1374	1412	-	cancer		Discrepancy Rules
9869602	<category="Disease Class">neoplasia</category>	1621	1666	-	neoplasia		
9949209	<category="Disease Class">inherited disorder</category>	276	331	-	inherited disorder		
9988281	<category="Modifier">tumour</category>	163	201	-	tumour		
9988281	<category="Disease Class">non-familial cancers</category>	348	404	-	non-familial cancers		
9988281	<category="Disease Class">sporadic cancers</category>	446	498	-	sporadic cancers		
9420335	<category="Specific Disease">gastrulation defect</category>	831	889	-	gastrulation defect		
9420335	<category="Specific Disease">gastrulation defect</category>	1010	1068	-	gastrulation defect		
9674906	<category="Specific Disease">early death</category>	924	974	-	early death		
9674906	<category="Specific Disease">congenital joint contractures</category>	1536	1604	-	congenital joint contractures		
9689113	<category="Specific Disease">von Willebrand factor (vWf) deficiency</category>	163	242	-	von Willebrand factor (vWf) deficiency		
9689113	<category="Specific Disease">vascular injury</category>	1108	1162	-	vascular injury		
9724771	<category="Specific Disease">metaplastic polyps of the colorectum</category>	2146	2221	-	metaplastic polyps of the colorectum		

9856499	<category="Specific Disease"> C7 defects</category>	574	623	-	C7 defects
9861003	<category="Specific Disease">hypomania</category>	463	511	-	hypomania
9867744	<category="Specific Disease">early death</category>	168	218	-	early death
9867744	<category="Specific Disease">iron overload</category>	573	625	-	iron overload
9888388	<category="Disease Class">neurological dysfunction</category>	556	616	-	neurological dysfunction
9888388	<category="Disease Class">gene defects</category>	2360	2408	-	gene defects
9931324	<category="Disease Class">congenital eye malformations</category>	103	167	-	congenital eye malformations
9931324	<category="Specific Disease">congenital absence of the iris</category>	260	329	-	congenital absence of the iris
9931324	<category="Disease Class">malformation of the eye</category>	350	409	-	malformation of the eye
9931324	<category="Specific Disease"> displaced pupils</category>	1410	1465	-	displaced pupils
9931324	<category="Specific Disease">searching gaze</category>	1534	1587	-	searching gaze
9931324	<category="Disease Class">PAX6 -related disease</category>	2014	2071	-	PAX6 -related disease
9949197	<category="Disease Class">cardiac defects</category>	1280	1331	-	cardiac defects
9949209	<category="Modifier">copper toxicosis</category>	23	71	-	copper toxicosis

9973276	<category ="Specific Disease">adenomatous polyps</category>	846	903	-	adenomatous polyps		
9457914	<category ="Specific Disease"> blistering</category>	421	470	-	blistering		
9457914	<category ="Specific Disease">nail dystrophy </category>	473	526	-	nail dystrophy		
9457914	<category ="Specific Disease"> patchy alopecia</category>	529	583	-	patchy alopecia		
9506545	<category ="Disease Class">Eye movement abnormalities</category>	0	62	-	Eye movement abnormalities		
9506545	<category ="Specific Disease">hypermetria </category>	883	933	-	hypermetria		
9506545	<category ="Specific Disease"> gaze-evoked nystagmus </category>	1348	1408	-	gaze-evoked nystagmus		
9506545	<category ="Specific Disease">hypometria </category>	1442	1491	-	hypometria		
9506545	<category ="Specific Disease"> gaze-evoked nystagmus </category>	1619	1679	-	gaze-evoked nystagmus		
9521325	<category ="Specific Disease">sudden death</category>	748	799	-	sudden death		
9585583	<category ="Specific Disease">coronal synostosis </category>	1080	1137	-	coronal synostosis		
9585583	<category ="Specific Disease"> brachycephaly</category>	1140	1192	-	brachycephaly		
9585583	<category ="Specific Disease">low frontal hairline</category>	1195	1254	-	low frontal hairline		

9585583	<category="Specific Disease">facial asymmetry</category>	1257	1312	-	facial asymmetry	The entity is a phenotype recorded in our reference databases and should not be annotated according to our annotation guidelines.	
9585583	<category="Specific Disease">ptosis</category>	1315	1360	-	ptosis		
9585583	<category="Specific Disease">hypertelorism</category>	1363	1415	-	hypertelorism		
9585583	<category="Specific Disease">broad great toes</category>	1418	1473	-	broad great toes		
9585583	<category="Specific Disease">clinodactyly</category>	1480	1531	-	clinodactyly		
9585606	<category="Specific Disease">premature death</category>	359	413	-	premature death		
9618170	<category="Specific Disease">skeletal myopathy</category>	514	570	-	skeletal myopathy		
9674906	<category="Specific Disease">myotonia</category>	372	419	-	myotonia		
9674906	<category="Specific Disease">skeletal dysplasia</category>	424	481	-	skeletal dysplasia		
9674906	<category="Specific Disease">campomelia</category>	742	791	-	campomelia		
9674906	<category="Specific Disease">skeletal dysplasia</category>	806	863	-	skeletal dysplasia		
9674906	<category="Specific Disease">hyperthermia</category>	1658	1709	-	hyperthermia		
9702690	<category="Disease Class">neuronal degeneration</category>	2164	2221	-	neuronal degeneration		
9709714	<category="Specific Disease">hepatomegaly</category>	658	709	-	hepatomegaly		

9709714	<category ="Disease Class">encephalopathy</category>	712	762	-	encephalopathy		
9709714	<category ="Specific Disease">hypotonia</category>	769	817	-	hypotonia		
9800909	<category ="Disease Class">cognitive impairment</category>	56	112	-	cognitive impairment		
9800909	<category ="Disease Class">cognitive impairment</category>	442	498	-	cognitive impairment		
9800909	<category ="Disease Class">cognitive impairment</category>	1172	1228	-	cognitive impairment		
9800909	<category ="Disease Class">cognitive impairment</category>	1594	1650	-	cognitive impairment		
9861003	<category ="Specific Disease"> mania</category>	409	453	-	mania		
9861003	<category ="Specific Disease"> depression </category>	541	590	-	depression		
9863607	<category ="Specific Disease">infertility</category>	355	405	-	infertility		
9867744	<category ="Specific Disease">cirrhosis</category>	694	742	-	cirrhosis		
9869602	<category ="Disease Class">neoplasia</category>	1621	1666	-	neoplasia		
9689113	<category ="Modifier ">vWf-deficient</category>	401	446	-	vWf-deficient		
9703418	<category ="Modifier ">C9-deficient</category>	474	518	-	C9-deficient		
9703418	<category ="Modifier ">C9-deficient</category>	746	790	-	C9-deficient		
9703501	<category ="Modifier ">deficient in BRCA1</category>	497	547	-	deficient in BRCA1	The entity is an adjective of a disease and should not be annotated according to [illegible]	

9463309	<category ="Modifier">hypohaptoglobinemic</category>	1051	1102	-	hypohaptoglobinemic	our annotation guidelines.	
9463309	<category ="Modifier">hypohaptoglobinemic</category>	1198	1249	-	hypohaptoglobinemic		
9856498	<category ="Modifier">C6-deficient</category>	1323	1367	-	C6-deficient		
9497246	<category ="Modifier">Breast Cancer</category>	138	183	-	Breast Cancer	The entity is a part of proper noun and should not be annotated according to our annotation guidelines.	
9497246	<category ="Modifier">Breast Cancer</category>	474	519	-	Breast Cancer		
9618166	<category ="Modifier">Pendred</category>	646	685	-	Pendred	The entity is not described fully and should not be annotated according to our annotation guidelines.	
9618166	<category ="Modifier">Pendred</category>	1165	1204	-	Pendred		
9689113	<category ="Modifier">von Willebrand</category>	547	593	-	von Willebrand		

The symbol of "-" indicates that we did not annotate the entity.
The "Pos_Start" and "Pos_End" were calculated by us, which were starting positions and ending positions of the "text" in the title and abstract of literature, because the corres

Species						
pmc	Pos_Start	Pos_End	Our Annotation	Experts' Annotation	Detailed Information	Classification
pmc1838407	21895	21905	-	Ivory-bill	The entity is not recorded in the NCBI_taxonomy database and should not be annotated.	Experts' False
pmc1891629	9288	9298	-	Haemogogus	The entity is classified as a genus in the NCBI_taxonomy database and should not be annotated.	
pmc2238978	2953	2960	-	anthrax	The entity is a disease according to the context of literature and should not be annotated as a species.	
pmc1075922	8962	8966	-	SV40	The entity is a species and should be annotated.	Our False
pmc2365090	25492	25498	-	bovine		
pmc1239921	20765	20770	-	Human	The entity is a part of proper noun and should not be annotated according to our annotation guidelines.	Discrepancy Rules
pmc2367462	1114	1119	-	Human		
pmc2367462	2771	2776	-	Human		
pmc2367462	3295	3300	-	Human		
pmc1937013	1047	1052	-	worms	The entity is the name of a type of species and should not be annotated according to our annotation guidelines.	
pmc1937013	22089	22094	-	worms		
pmc1937013	23018	23023	-	worms		
pmc1937013	25005	25010	-	worms		
pmc1937013	28385	28390	-	worms		
pmc1937013	39439	39444	-	worms		
pmc1937013	40131	40136	-	worms		
pmc1937013	46241	46246	-	worms		
pmc1937013	46330	46335	-	worms		
pmc1937013	46557	46562	-	worms		
pmc1937013	48630	48635	-	worms		
pmc1937013	48690	48695	-	worms		
pmc1937013	49082	49087	-	worms		
pmc1937013	49383	49388	-	worms		
pmc150219	9967	9973	-	salmon		

The "-" indicates that we did not annotate the entity.
The "Pos_Start" and "Pos_End" were provided by the corresponding corpus.

Stable 3. The performance comparison (F1 values) of our Phase I BERT-based NER model and PTC, Hunflaire, BERN and BERN2

Dataset	Entity	PTC	Hunflaire	BERN	BERN2	BERT (Phase I)
**BC2GM**	**Gene/ protein**	78.8	77.9	83.4	83.7	84.4
**NCBI-disease**	**Disease**	81.5	85.4	88.3	88.6	89.7
**tmVar2**	**Mutation**	93.7	N/A	93.7	93.7	93.9
**Linnaeus**	**Species**	85.6	93.2	88.0	92.7	93.1

Note: F1 scores were reported. The F1 scores of variant extraction tools — PTC, BERN and BERN2 are the same because they shared the tmVar2 tool.

Stable 4. the performance comparison of BERT-based NER in the Phase I finetuning steps for gene, variant, disease and species at the entity level in term of joint and single entity extraction methods

Entity Type	Entity Extraction Method	BERT Phase I		
		Precision (%)	Recall (%)	F1-Score (%)
**Gene**	**Joint**	87.56	88.25	87.9
	**Single**	88.07	88.12	88.1
**Variant**	**Joint**	85.76	98.15	91.54
	**Single**	87.24	98.24	92.42
**Disease**	**Joint**	90.17	91.64	90.9
	**Single**	90.28	91.14	90.71
**Species**	**Joint**	97.20	98.42	97.81
	**Single**	97.52	98.37	97.94

Stable 5. The performance comparison (F1 values) of our Phase I DistilBERT-based NER model and DistilBERT, DistilBioBERT, CompactBioBERT and TinyBioBERT

Dataset	Type	DistilBERT	DistilBioBERT	CompactBioBERT	TinyBioBERT	DistilBERT (Phase I)
**BC2GM**	**Gene/ protein**	84.61	86.60	86.71	82.52	83.51
**NCBI-disease**	**Disease**	86.38	87.93	88.67	85.22	87.98
**BC4CHEMD**	**Drug/chemical**	89.53	91.77	91.40	89.03	90.43
**Linnaeus**	**Species**	80.73	83.29	82.90	78.29	88.62

Note: F1 scores were reported.
